# Invisible Active Bleeding Due to the Watershed Phenomenon

**DOI:** 10.5334/jbsr.3604

**Published:** 2024-05-23

**Authors:** Miloud Dewilde, Birgitt Janssens

**Affiliations:** 1 Schaapsdreef 50a, 8500 Kortrijk, Belgium; 2 UZ Leuven

**Keywords:** Watershed phenomenon, veno-arterial extracorporeal membrane oxygenation (VA-ECMO), heart failure, CT angiography (CTA), active bleeding, abdominal

## Abstract

A case is presented of an 83-year-old female patient with a strong suspicion of active bleeding, but no diagnostic contrast blush could be seen on the original computed tomography (CT) scan.

*Teaching point:* When performing CT angiography in veno-arterial extracorporeal membrane oxygenation (VA-ECMO), it is important to understand the altered haemodynamics, as flow-related artefacts such as the vascular watershed phenomenon can obscure bleeding.

## Introduction

In patients receiving peripheral veno-arterial extracorporeal membrane oxygenation (VA-ECMO) support, the term vascular watershed describes the phenomenon that blood coming from the ECMO has an opposite direction than blood coming from the left ventricle [1] and thus creates blurring at the level where the two streams intermingle with transition from dens contrast to virtually no contrast in the distal portion.

## Case Report

An 83-year-old female underwent elective mitral and tricuspid valve plasty, initially via Heartport access, but complicated perioperatively by right ventricular bleeding (after insertion of pacing wires), for which conversion to sternotomy was required. While transferring from the operating table to the hospital bed, a haemodynamic collapse occurred, necessitating urgent placement of VA-ECMO. The hypovolaemic shock was caused by bleeding from the right superficial femoral artery, for which surgical exploration and suturing were performed. The patient was admitted to the intensive care unit.

Given the patient’s persistent need for fluid resusci-tation despite efforts to stabilise the vital parameters, a thoracoabdominal CT angiography (CTA) was conducted, extending to the mid-thigh, to rule out persistent active bleeding.

**Figure 1:**
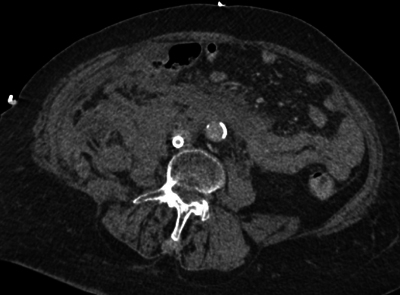
An axial CT of extensive intra- and retroperitoneal blood at the level of the infrarenal aorta.

**Figure 2:**
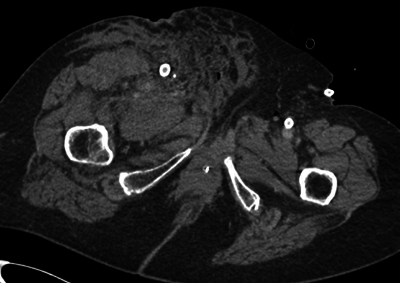
An axial CT showing extensive inguinal blood at the level of the right superficial femoral artery.

The primary CTA showed extensive intra- and retroperitoneal haemorrhage (Figure 1), as well as in the right groin (Figure 2). No active bleeding could be visualized. However, there was a clinically strong suspicion of active bleeding at the level of the right groin, yet without demonstrable contrast extravasation.

**Figure 3:**
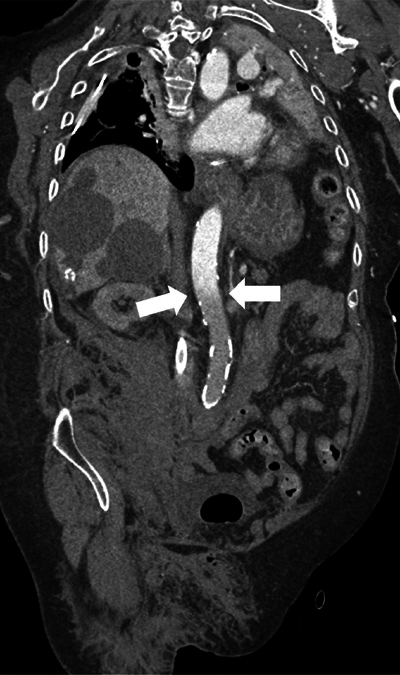
Paracoronal reformatted image of the watershed zone (white arrows) at the level of the superior mesenteric artery (SMA).

**Figure 4:**
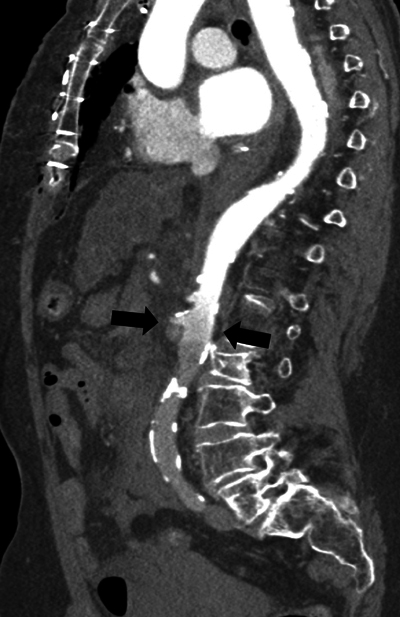
Parasagittal reformatted image of the watershed zone (black arrows) at the level of the superior mesenteric artery (SMA).

**Figure 5:**
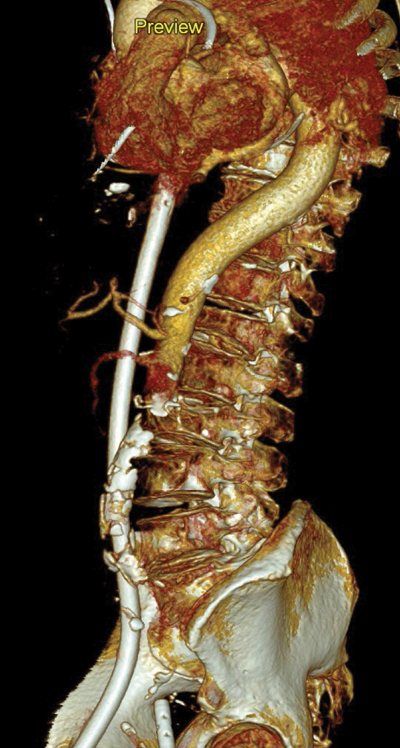
3D reconstruction of the watershed zone at the level of the superior mesenteric artery (SMA).

CTA demonstrated good opacification of the thoracic aorta up to the level of the superior mesenteric artery (SMA). However, more distally, no marked enhancement of the aorta and visceral vessels was obtained (Figures 3–5).

Given the suspicion of active haemorrhage in the abdomen and right groin, yet with persistent uncertainty on the precise location and considering the patient’s limited cardiac function, the CT scan was promptly repeated during the brief discontinuation of the VA-ECMO support. This was supported by a team consisting of a perfusionist, anaesthesiologists and intensive treatment unit (ICU) nurses.

**Figure 6:**
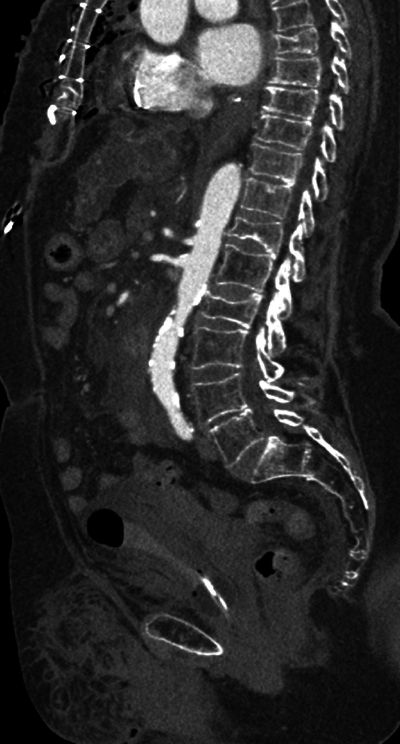
Parasagittal image of the contrast-enhanced aorta.

**Figure 7:**
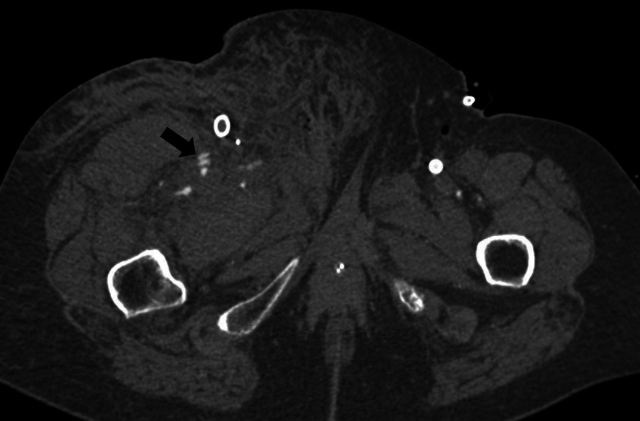
An axial image of a clear arterial contrast blush from the superficial femoral artery and a side branch.

On the repeat CTA, the entire aorta was enhanced well (Figure 6), and a clear arterial contrast blush was observed originating from the superficial femoral artery and a side branch (Figure 7).

## Discussion

In this case, no luminal contrast opacification nor active haemorrhage distal to the SMA was present due to the watershed phenomenon, with the blurred area at the level of the SMA and despite the haemorrhage surrounding the right superficial femoral artery.

Since distal to the SMA no vascular enhancement was obtainable, active bleeding could not be ruled out. Awareness of the watershed phenomenon is crucial, since artefacts can be mistaken for pseudolesions such as thrombus, dissection, or vascular occlusion. The watershed area can also potentially obscure true pathology [2], with potentially fatal consequences, as in this case where an active bleeding could be overlooked.

Therefore, it is crucial to know the position of the ECMO cannula when performing and reporting a contrast-enhanced CT scan of a patient under ECMO.

Since this patient still had moderate residual cardiac function, the watershed zone was at the level of the SMA, yet she was prone to cardiac and brain ischemia since oxygenated blood could not reach the carotids or heart.

Often, ischemia occurs in the brain and/or heart. As the patient recovers, the strength of the heart increases, thus preventing the oxygenated blood from the ECMO cannula to reach the brain or heart.

Blood gases obtained from the right radial artery reflect the oxygenated blood delivered to the brain but do not necessarily reflect the oxygen content delivered to the coronary arteries.

The decision to briefly take off the ECMO and rely shortly on the cardiac output caused an arterial blush to emerge, thus enabling the demonstration of contrast extravasation with certainty and refer for targeted therapy.

## Conclusion

In VA-ECMO, the peripheral placement of the ECMO-cannula provokes haemodynamic alterations with retrograde arterial blood flow, creating a watershed phenomenon at the level of flow collision. A watershed phenomenon can obscure active bleeding, thrombus, dissection or vascular occlusion. Adjusting the CT procedure to the clinical question is crucial for patients with an ECMO cannula. In this case, active bleeding of the superficial femoral artery became visible only after discontinuation of ECMO in the presence of a supporting multidisciplinary team.
